# Physiology and Pathology the True Foundation for Medical Practice

**Published:** 1864-04

**Authors:** John Hughes Bennett


### SELECTED.

### PHYSIOLOGY AND PATHOLOGY THE TRUE FOUNDATIONS FOR MEDICAL PRACTICE.

EXTRACT FROM A LECTURE

By JOHN HUGHES BENNETT, M. D.

When we investigate closely into what is actually known of our therapeutical means, divided into alimenta, hygienica, and materia medica, it will be seen that we have no exact details founded on scientific research. What we require is, that such details must be first arrived at, and then applied in accordance with pathological laws. These point out that all treatment must be general and special—general as regards the nature of the disease, special as regards its seat. The great problem in conducting any given case is to carry out both indications, so that one does not interfere with the other. If, for example, the object be to favor the removal of inflammation or tubercle from the lung, the means requisite for that end must not be put aside or counteracted by a desire of alleviating pain, breathlessness, or expectoration. Indeed, one point of great importance, and which clinical observation has in recent times made manifest, is, that general and local symptoms frequently bear no relation whatever to the fatality of the lesion. Thus, an extensive acute inflammation of the lungs, a febricula, or an impacted gall-stone, may cause the most violent symptoms and perturbation of the economy, and yet spontaneously terminate in recovery in a few days; while a phthisis, a pleurisy with effusion, or even a pneumothorax, which may permanently destroy the action of a lung, may come on imperceptibly, and cause only trifling functional symptoms. To the pathologist, therefore, such symptoms are no longer the same guides to treatment as they used to be. They do not so much excite his regard as the causes or structural and chemical lesions which produce them, for he knows that the former will disappear if the latter are removed. It need not, therefore, excite surprise that as our knowledge of pathology has advanced, and our means of diagnosis have improved, we direct our attention more to the morbid alteration and less to the temporary effects. In this way it has gradually become manifest that so far from doing good by attempts to relieve symptoms, we positively do harm to the disease. If, for instance, impaired digestion cause headache and sleeplessness, the relief of these symptoms by morphia is anything but beneficial, inasmuch as it depresses the nervous system and diminishes the appetite, and so increases the real disease. For the same reason, of what advantage can sedatives and cough mixtures be in phthisis? The true indication for treatment is to strengthen the appetite, increase the nutrition, aud invigorate the frame. Medicines which only temporarily lull irritation, create nausea, destroy appetite, and favor diaphoresis, however they may relieve symptoms, can never arrest the disease.

Not long ago a young American physician brought under my notice a tincture of the veratrum viride, which he maintained possesses the power of diminishing the force of the pulse, and said that on this account it was a most valuable medicine in fevers, inflammations, and other diseases where the pulse was excited. But pathology indicates that so far from lowering the pulse in these disorders, what is required is in truth to support it, for the reasons I have formerly mentioned. Indeed, I can not conceive any circumstances in which such a remedy, even if it possessed the virtues ascribed to it, can be useful. But it so happens that several years ago Dr. Norwood, of Nashville, in the United States, sent me a bottle of the tincture, which I tried in several cases of fever in the infirmary. In every instance the medicine caused violent vomiting, pain in the stomach, weak pulse, and symptoms of collapse, and had to be discontinued; but in no one instance did it shorten the disease or improve the symptoms — quite the contrary. Yet this remedy is once more recommended to us on the ground of subduing, not a disease, but a symptom, although everything we know of pathology and the natural history of fevers and inflammations is entirely opposed to its employment.

In the same manner, hosts of new drugs, or new preparations of old ones, are constantly extolled and recommended on the most insufficient data, no one seeming to think it necessary to make experiments, careful observations, or deductions, but appealing only to a very limited experience. But we have previously seen that even where experience has been universal and unanimous—as in the case of bloodletting in inflammations—what mischief and error have arisen from unacquaintance with physiology and pathology.

As another example, let us for a moment consider the contradictory opinions that prevail with regard to a medicine which, perhaps, has been more extensively tried than any other; I allude to mercury. I need not cite the extravagant praises which it has received from its partizans. It will suffice to say, that the most accomplished professor of materia medica in these times tells us that, physiologically, it is "a corrosive, irritant, errhine, cathartic, and astringent; a stimulant, diuretic, diaphoretic, cholagogue, and emmenagogue; and an excitor of that peculiar state of the constitution denominated mercurial action, of which salivation is one of the chief local signs. Therapeutically," he says, "it is antiphlogistic, alterative, sedative or contra-stimulant, deobstruent, antisyphilitic, and anthelmintic." (Christison.) A drug possessed of such wonderfully extensive and varied powers should certainly by this time have had its virtues universally recognized; yet the fact is that, with the exception of its action as a sialagogue and a cathartic, there is scarcely one other of its supposed virtues that is not disputed.

Is mercury a cholagogue? We have no proof whatever that it increases the secretion of bile; and the only experimental investigation with which I am acquainted—viz.: that of Dr. Scott, who gave calomel to dogs, and then collected the bile through a fistulous opening made into the biliary duct, found it in three days to diminish the quantity of that fluid.* Is it an antisyphilitic? In recent times it is admitted that syphilis has diminished in intensity just in proportion as the use of mercury has declined; and the gigantic experiments made on entire garrison regiments in France, Germany and Sweden, prove that the non-mercurial treatment of syphilis is far superior to the mercurial in every respect. Is it antiphlogistic? All that we know of modern practice negatives the idea. Does it cause absorption of lymph or the coagulated exudation? The clinical observations of Prof. John Taylor, of London, in pericarditis, and of Dr. Williams, of Boston, United States, in irritis, are opposed to such a supposition.

Then as to its mode of administration what differences exist. Some give it in large, others in small doses—some in acute, others in chronic diseases of the same kind. Some argue that it should precede, others follow venesection. Some combine calomel with blue pill to intensify its action; others with opium for the same reason. Its applications are so numerous and contradictory, that the question may well be, not for what dis-

*Beale's Archives of Medicine, vol. i, p. 269.

eases is it useful, but rather, which has *not* been represented to be benefited by this drug? In the meantime, it has been admitted that it arrests the appetite, checks nutrition, excites a peculiar fever and erethism, produces a coppery taste in the mouth, furred tongue, and salivation; and the pathologist may well inquire how a poison operating in such a way can have any curative tendency whatever?

Now why all this uncertainty as to the therapeutic action of drugs? My answer is—In consequence of our ignorance of an exact diagnosis and of a true pathology. Many persons think that the science of therapeutics is to be advanced by trying the effects of drugs on animals, by clinical observation, by records of cases, and so on; but whatever amount of knowledge may be thus arrived at, it can never be advantageous for medical treatment, until, as I have endeavored to show, we are capable of recognizing with exactitude the disease we investigate, and, secondly, know its nature and natural progress.

These steps must be preliminary to all advance in therapeutics, and that they have not hitherto been made so, is at once the explanation of past failure, and the indication for future success. The true promoters of therapeutics, consequently, are not those men who pass their lives in treating patients as well as they can from the results of pre-existing or present knowledge; they are not those who are constantly arranging the opinions and assertions of former writers as to the effects of past treatment; but they are those who direct all their energies to improving diagnosis and advancing physiology and pathology. This conviction must force itself on the minds of all who seriously consider the subject, and, in truth, it is the one which renders every earnest and truthful student amongst us a pathologist. The result is already obvious. We are gradually sweeping away the errors of empiricism, slowly clearing the ground for the erection of a more simple and solid temple of knowledge. This accomplished, we hope to accumulate, by laborious toil in research, materials for its foundation,—a work to which I think we are gradually approaching,—in the hope that, by patience and perseverance, a day will arrive when Medicine will be generally allowed to have approximated towards, if it does not actually reach, the character of an exact science.

The true principles, therefore, which should guide our efforts to advance therapeutics are—

1st. That an empirical treatment derived from blind authority, and an expectant treatment originating in an equally blind faith in nature, are both wrong.

2nd. That a knowledge of physiology and pathology is the real foundation and necessary introduction to a correct study of therapeutics.

3rd. That a true experience can only have for its proper aim the determination of how far the laws evolved during the advance of these sciences (physiology, pathology and therapeutics) can be made available for the cure of diseases.

